# Mesonephric adenocarcinoma with a sarcomatous component, a notable subtype of cervical carcinosarcoma: a case report and review of the literature

**DOI:** 10.1186/1746-1596-8-74

**Published:** 2013-05-07

**Authors:** Shiori Meguro, Masanori Yasuda, Michio Shimizu, Akira Kurosaki, Keiichi Fujiwara

**Affiliations:** 1 Departments of Pathology, Saitama Medical University International Medical Center, 1397-1 Yamane, Hidaka, Saitama, 350-1128, Japan; 2 Departments of Gynecologic Oncology, Saitama Medical University International Medical Center, 1397-1 Yamane, Hidaka, Saitama, 350-1128, Japan

**Keywords:** Mesonephric adenocarcinoma, Carcinosarcoma, Uterine cervix

## Abstract

**Abstract:**

Carcinosarcoma of the uterine cervix is less common than its counterpart in the uterine corpus. On the other hand, mesonephric adenocarcinoma is also a rare neoplasm in the uterine cervix, and it has been reported that mesonephric adenocarcinomas are often accompanied by sarcomatous components. We present a case of mesonephric adenocarcinoma with a sarcomatous component which arose in a 63-year-old postmenopausal woman. The hysterectomy specimen grossly showed an exophytic mass measuring 1.8 cm in the uterine cervix. Histologically, diffuse mesonephric hyperplasia and adenocarcinoma with malignant spindle cell proliferation was recognized, and therefore the tumor was diagnosed as “mesonephric adenocarcinoma with a sarcomatous component.” The review of the literature of cervical carcinosarcoma and cervical mesonephric adenocarcinoma revealed that 16% of cervical carcinosarcoma is of mesonephric duct origin, and that mesonephric adenocarcinoma seems to be more likely to have sarcomatous change. We think the presence of a sarcomatous component in the cervical biopsy specimen could be helpful in the diagnosis of mesonephric duct origin.

**Virtual slides:**

The virtual slide(s) for this article can be found here: http://www.diagnosticpathology.diagnomx.eu/vs/1243996503911062

## Background

Carcinosarcoma is the neoplasm composed of malignant epithelial and mesenchymal components, and a mesenchymal component of carcinosarcoma is recently considered as a metaplastic change of carcinoma. Carcinosarcoma arises less frequently in the uterine cervix and the vulva, than in the uterine corpus [1,2]. Although not much attention has been paid to it, cervical carcinosarcoma can be characterized by having two different origins: the Müllerian ducts and the mesonephric duct remnants [3-7]. Mesonephric adenocarcinoma arises from mesonephric duct remnants. Unlike the usual endocervical-type adenocarcinoma, it is known that mesonephric adenocarcinoma is not related to human papillomavirus (HPV) infection [5,8-10]. We present a case of mesonephric adenocarcinoma with a sarcomatous component arising from mesonephric hyperplasia of the uterine cervix, and comprehensively review the literature of the cervical carcinosarcoma arising from Müllerian ducts and mesonephric ducts.

## Case presentation

A 63-year-old, Japanese postmenopausal woman with abnormal vaginal bleeding consulted our hospital. MR imaging showed an enhanced uterine cervical mass measuring 2.0 cm in diameter. There was no evidence of lymphadenopathy or metastasis. The cervical biopsy specimen showed diffuse malignant spindle cell proliferation with myxoid stroma. The tumor cells were focally arranged in small nests or glands, resulting in a diagnosis of undifferentiated carcinoma. The patient underwent radical hysterectomy, bilateral salpingo-oophorectomy, and pelvic lymphadenectomy, without adjuvant chemotherapy. Seven months after the operation, the patient was treated by chemoradiative therapy because of local recurrence, and the recurrent tumor disappeared after three months of the therapy.

**Figure 1:**
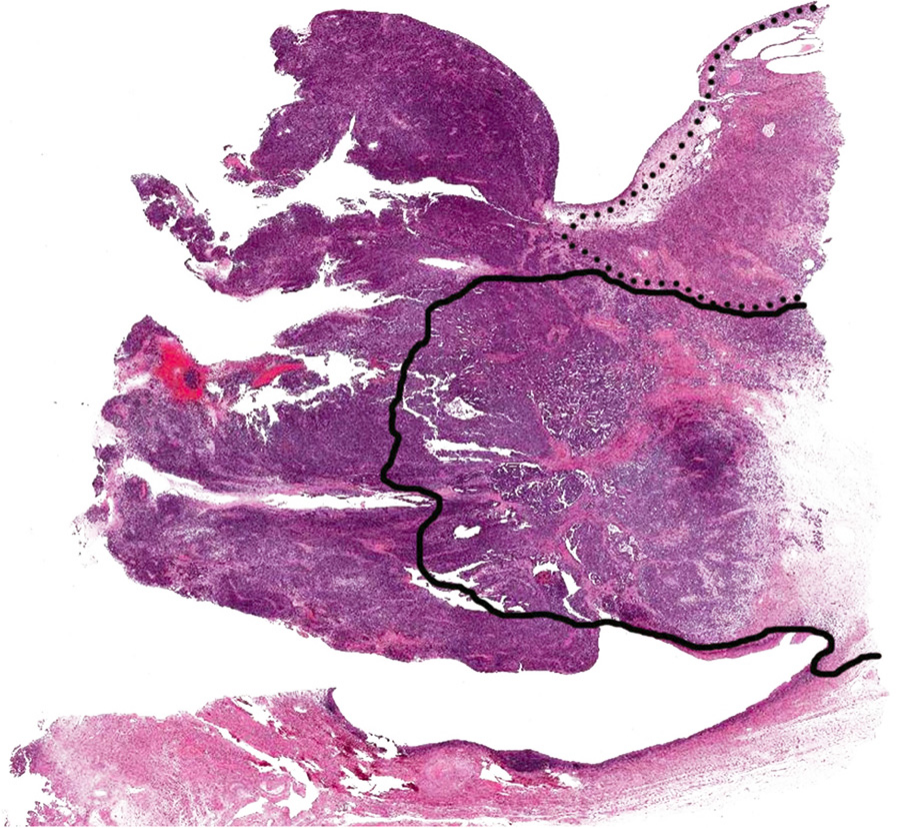
**Loupe view of the uterine cervical tumor.** The tumor showed exophytic polypoid growth. The area of hyperplasia (enclosed by the dotted line) covered about 20% of the lesion, and the adenocarcinoma area (solid line) covered about 40%. The carcinosarcoma component (unenclosed area of the polypoid lesion) accounted for about 40%.

**Figure 2:**
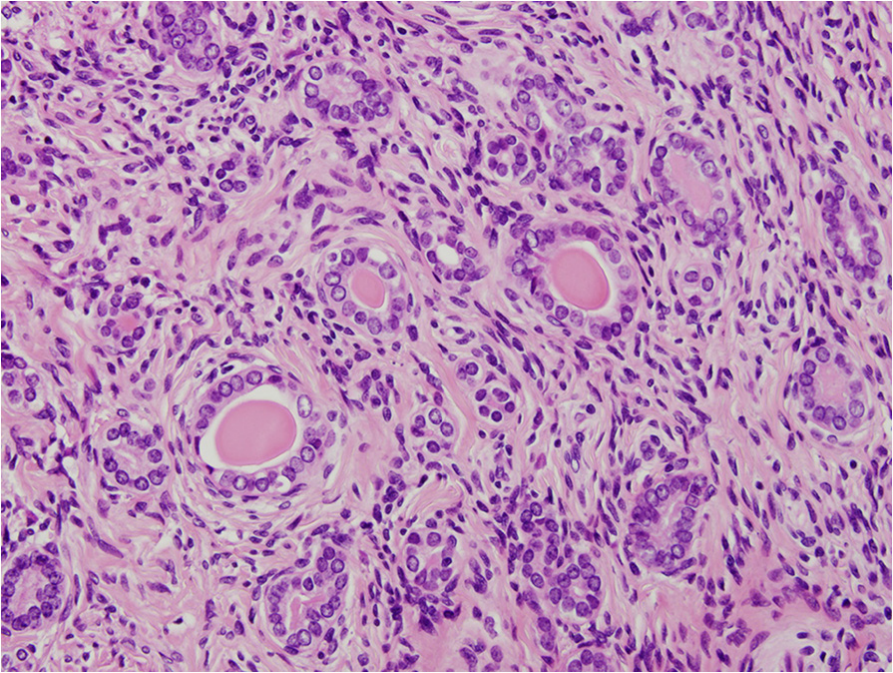
**The microscopic view of mesonephric hyperplasia.** In mesonephric hyperplasia, the cytological atypia was bland and mitotic figures were rarely seen.

**Figure 3:**
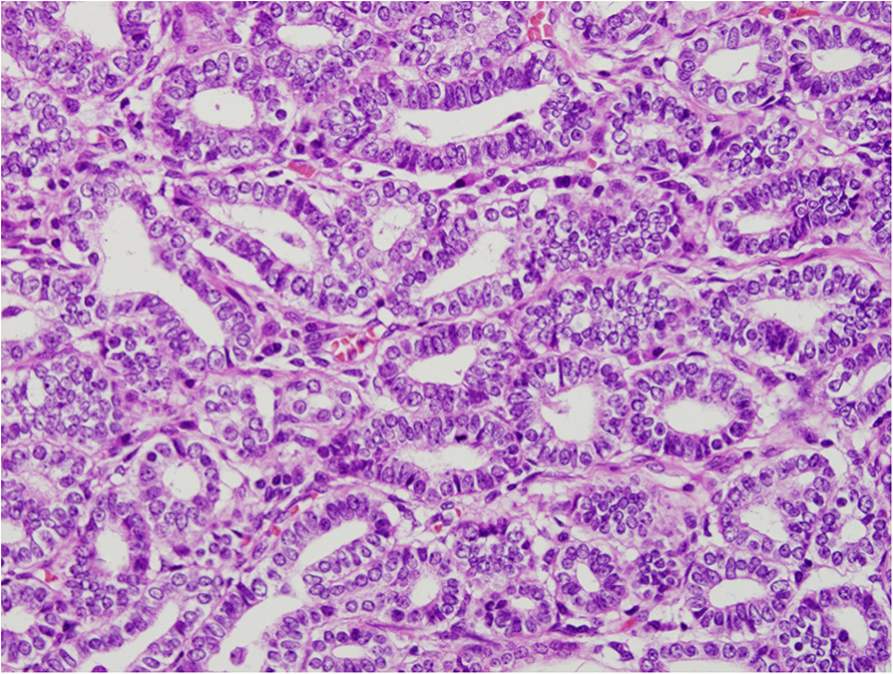
**Mesonephric adenocarcinoma in the adjacent area of mesonephric hyperplasia.** The adenocarcinoma cells were arranged as small irregular glands.

**Figure 4:**
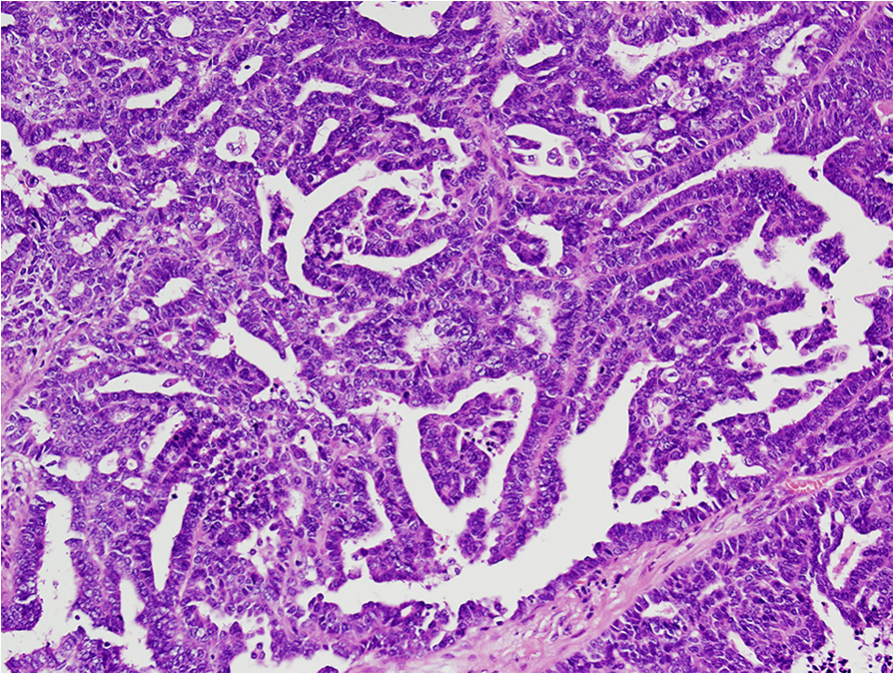
**Mesonephric adenocarcinoma with papillary and ductal structures.** Papillary and ductal patterns were also observed in a part of the adenocarcinoma component.

**Figure 5:**
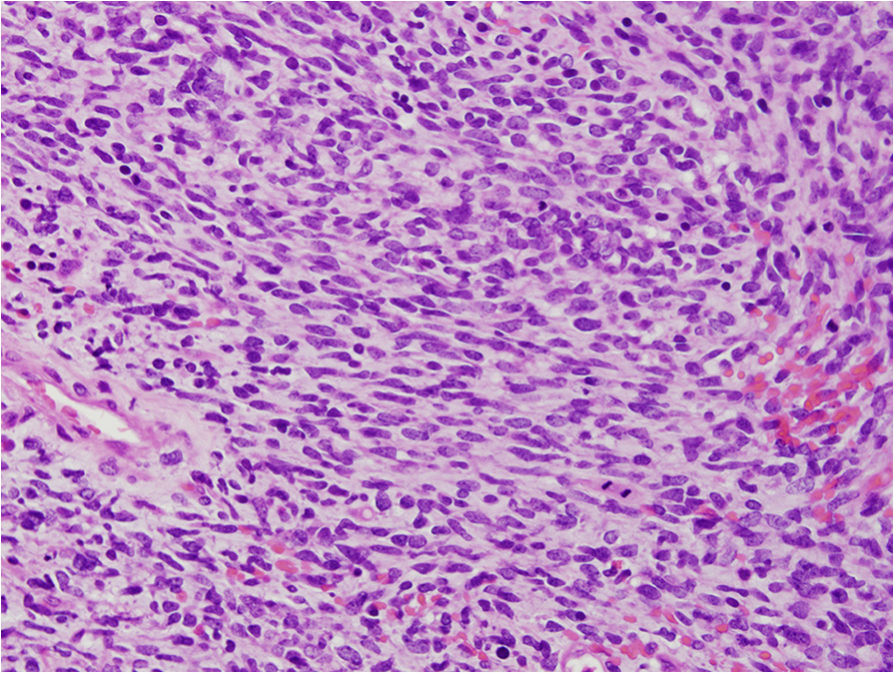
**The sarcomatous component.** The sarcomatous component was composed of spindle cell proliferation.

**Figure 6:**
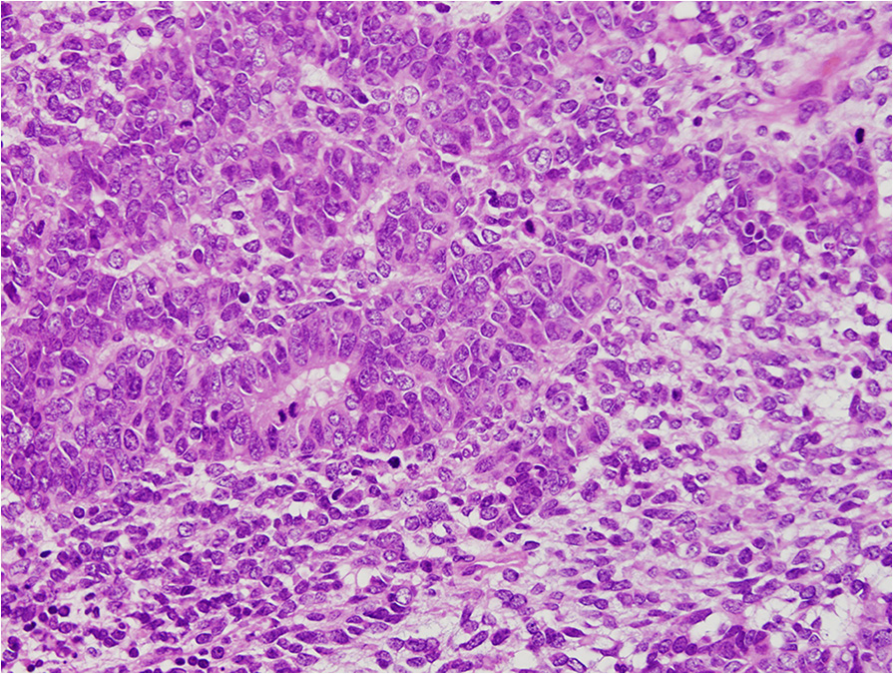
A transition between the carcinomatous component and the sarcomatous component.

The hysterectomy specimen grossly showed an exophytic mass in the left lateral wall of the cervix, measuring 1.8 cm (Figure 1). Microscopically, mesonephric hyperplasia of the diffuse type (Figure 2) extended to the entire circumferences of the cervix, and a small number of mesonephric tubules were found in the vagina and myometrium. In the adjacent area of mesonephric hyperplasia, adenocarcinoma composed of small irregular glands was observed. Compared to hyperplasia, these irregular glands had severe cytological atypia (Figure 3). Papillary pattern and ductal pattern were also present (Figure 4). Furthermore, a sarcomatous component composed of polygonal and spindle-shaped cells was recognized (Figure 5), and there was a transition from the adenocarcinomatous component to the sarcomatous component (Figure 6). No heterologous component was noted. The sarcomatous component was interpreted as undifferentiated carcinoma as in the previous biopsy specimen. The results of the immunohistochemical study are shown in Table 1. Based on these findings, the diagnosis of mesonephric adenocarcinoma with a sarcomatous component arising in mesonephric hyperplasia was finally confirmed, being staged at pT2a because of minimal invasion of the adenocarcinoma into the vaginal wall.

**Table 1:** Antibodies used for immunohistochemical staining and its results

**Antigen**	**Antibodies**			**Results**		
	**Clone**	**Source**	**Dilution**	**Biopsy**	**Hysterectomy**	
					**Adenoca.**	**Sarscomatous component**
AE1/AE3	AE1/AE3	Dako, Glostrup, Denmark	1: 50	+	+++	-
CAM5.2	CAM5.2	BD Biosciences, California, USA	1: 2	ND*	+++	-
vimentin	V9	Dako	1: 100	+++	-	+++
ER	SP1	Roche Diagnostics, Basel, Switzerland	1: 1	-	-	-
PgR	1E2	Roche Diagnostics	1: 1	-	-	-
AR	AR441	Dako	1: 50	ND*	-	-
CD10	56C6	Leica Microsystems, Newcastle, UK	1: 40	-	++	++
calretinin	5A5	Leica Microsystems	1: 100	-	+	-

ND*, not done.

Immunopositivity was semi-quantitatively assessed as follows: 3+, more than 10% of the tumor cells positive; 2+, 1-10% of the tumor cells positive; 1+, less than 1% tumor cells positive; no tumor cell staining was considered negative.

## Discussion

The mesonephric ducts run parallel to the Müllerian ducts in the fetus, and regress as female development progresses [11]. Mesonephric remnants are detected by chance in the lateral wall of the vagina and uterus, the broad ligament, the mesosalpinx, or the ovarian hilus, and occur at a frequency of about 20% in the adult uterine cervix [12]. The remnants sometimes become hyperplastic, and rarely mesonephric adenocarcinoma can develop with the background of remnants or hyperplasia.

The present case was characterized by the presence of a sarcomatous component. In the biopsy specimen, because only a spindled cell component was obtained, the tumor was initially diagnosed as “undifferentiated carcinoma.” In the previous literature, differential diagnoses of mesonephric adenocarcinoma included clear cell adenocarcinoma, endometrioid adenocarcinoma, serous adenocarcinoma, usual endocervical-type adenocarcinoma, and minimal deviation adenocarcinoma [5,13]. However, it was not until recently that attention has been paid to the presence of a sarcomatous component as a helpful tool in the differential diagnosis of mesonephric adenocarcinoma.

Including the present case, 42 cases with mesonephric adenocarcinoma of the uterine cervix have been reported in the English literature. Ten out of the 42 cases (24%) had a sarcomatous component (Table 2) [3-7]. Regarding these 10 cases, the mean occurrence age was 54 years; tumor size ranged from 1.8 to 8.0 cm (mean 4.5) in the largest diameter; and 4 of the tumors were grossly exophytic or polypoid lesions [3,4,7]. The sarcomatous components were as follows: spindle cell sarcoma in 6 cases [3-5]; osteosarcoma in 2 cases [3,6,7]; chondrosarcoma in 1 case [4]; rhabdomyosarcoma in 1 case [4].

**Table 2:** Mesonephric tumors with a sarcomatous component

**Case**	**Source (ref no.)**	**Age (year)**	**Size (cm)**	**Pathological diagnosis**	**Heterologous component**	**FIGO stage**	**Outcome**
1	Present case	63	1.8	Mesonephric adenoca. with spindle cell component	No	IIA	NED 10 mo
2	3	37	ND	Mesonephric adenoca. with spindle cell component	No	IB	AWD 11 yr
3	3	40	ND	Mesonephric adenoca. with spindle cell component	No	IB	NED 2.3 yr
4	3	73	ND	Mesonephric adenoca. with spindle cell component	No	IB	NED 3 yr
5	4	54	6	Malignant mixed mesonephric tumor	No	IIA	DOD 7 mo
6	5	ND	ND	Mesonephric adenoca. With spindle cell component	No	ND	ND
7	3, 6	39	ND	Mesonephric adenoca. with spindle cell component	Osteosarcoma	IB	DOD 6.2 yr
8	7	65	3	Osteosarcoma associated with mesonephric rests	Osteosarcoma	IB	NED 1 yr 11mo
9	4	54	3.5	Malignant mixed mesonephric tumor	Chondrosarcoma	IB	NED 1 yr 1mo
10	4	62	8	Malignant mixed mesonephric tumor	Rhabdomyosarcoma	IVB	AWD 3 yr 3mo

ND, not described; adenoca., adenocarcinoma; NED, no evidence of disease; AWD, alive with disease; DOD, dead of disease.

On the other hand, including this case, 61 cases of cervical carcinosarcoma have been reported in the English literature. Of these, 10 cases (16%) were of mesonephric duct origin, and 51 cases (85%) were of Müllerian duct origin, traditionally designated as malignant Müllerian mixed tumor (MMMT). Regarding the epithelial component of MMMT, adenocarcinoma and squamous cell carcinoma were recognized with approximately the same frequency. Basaloid carcinoma, adenoid basal carcinoma, and adenoid cystic carcinoma were also observed in a few cases. Carcinosarcoma is an extremely rare tumor of the uterine cervix. However, from the point view of mesonephric duct origin, one-quarter of them have a sarcomatous component, and they account for 16% of cervical carcinosarcoma. These values may represent an overestimate since these are more likely than the Müllerian variants to be reported in the literature. Even so, interestingly, it seems that mesonephric adenocarcinoma is more likely to have sarcomatous change than the usual uterine cervical carcinoma arising from Müllerian ducts.

In the present case, the cervical tumor was initially diagnosed as “undifferentiated carcinoma” in the biopsy. Mesonephric adenocarcinomas are usually difficult to make a correct diagnosis by small specimen of biopsy because of their common occurrence site of the deep and lateral wall of the uterine cervix. In addition, mesonephric adenocarcinomas are known immunohistochemically to express CD10, calretinin, and androgen receptor [4,6,13,14], but these immunohistochemical profiles have not been evaluated enough in the sarcomatous component. Considering that 16% of cervical carcinosarcoma is of mesonephric duct origin, if a sarcomatous component is recognized in the biopsy specimen, it may be suspected of being of mesonephric duct origin.

As with cervical squamous cell carcinoma, the association between cervical adenocarcinoma of the usual type and HPV infection is well known [8,9,15-17]. However mesonephric adenocarcinoma is not related to HPV infection [5,8-10]. Although an HPV test was not performed in the present case, the tumor is predicted to be HPV-negative.

Looking at Table 2, 2 cases (22%) died [3,4,6]; 2 cases (22%) were alive with metastatic or local recurrent tumor [3,4]; and 5 cases (56%) were free of disease [3,4,6]. In contrast, in the cases of mesonephric adenocarcinoma without a sarcomatous component, 20% of cases died of the disease [6,18-20]. It seems that there is no significant difference in the prognosis between mesonephric adenocarcinoma with and without a sarcomatous component, although it has been previously reported that mesonephric adenocarcinoma with a sarcomatous component showed aggressive behavior [4,21].

## Conclusion

We presented a case of mesonephric adenocarcinoma with a sarcomatous component arising from mesonephric hyperplasia of the uterine cervix. It seems that mesonephric adenocarcinoma is more likely to have sarcomatous change. Mesonephric adenocarcinomas are difficult to make a correct diagnosis by small biopsy specimens; however, the presence of a sarcomatous component could be helpful in the diagnosis of mesonephric duct origin. Regarding the relation between the sarcomatous component and prognosis, further studies are needed.

## Consent

Written informed consent was obtained from the patient for publication of this Case Report and any accompanying images. A copy of the written consent is available for review by the Editor-in-Chief of this journal.

## Competing interests

The authors declare that they have no competing interests.

## Authors’ contributions

SM drafted the manuscript. MY conducted the reporting method of the present case. MS helped to draft the manuscript. AK and KF analyzed the clinical information as gynecological oncologists. All authors read and approved the final manuscript.
